# Recurrent COVID‐19 infection in a case of rituximab‐induced hypogammaglobulinaemia

**DOI:** 10.1002/rcr2.891

**Published:** 2021-12-07

**Authors:** Jefferson Daniel, Balamugesh Thangakunam, Barney Thomas Jesudason Isaac, Mahesh Moorthy, Devasahayam Jesudas Christopher

**Affiliations:** ^1^ Department of Pulmonary Medicine Christian Medical College Vellore India; ^2^ Department of Clinical Virology Christian Medical College Vellore India

**Keywords:** COVID‐19, hypogammaglobulinaemia, recurrent COVID‐19, re‐infection, rituximab

## Abstract

Patients with immunodeficiency are at an increased risk of recurrent COVID‐19 infection. They may lack the natural immune response that usually confers long‐lasting immunity. Here, we present our experience managing one such patient, who had a COVID‐19 infection twice, 5 months apart. He had a positive SARS‐CoV‐2 real‐time reverse transcription polymerase chain reaction (RT‐PCR) and computed tomography (CT) thorax with classical findings of COVID‐19 on both occasions. He had multiple negative RT‐PCR tests and two CT scans without COVID‐19 features between these two infections. While the antibody response to the first infection was not detectable, the response to the second infection was robust. Live attenuated vaccines are contraindicated in patients with immunodeficiency, and other vaccines may not elicit an adequate immune response. A high index of suspicion for recurrent COVID‐19 is warranted in this group of patients.

## Graphical abstract

Recurrent COVID‐19 should be considered while managing post‐COVID complications. Patients with immunodeficiency are at an increased risk of COVID‐19 re‐infection.

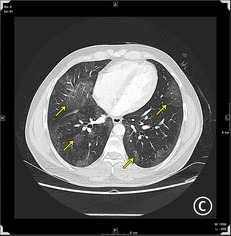

## INTRODUCTION

Re‐infections with SARS‐CoV‐2 are being reported lately. Three commonly proposed scenarios are persistent detection of residual virus after COVID‐19 recovery, false‐negative test between two true‐positive tests and true re‐infection.^1^ There is a general concern over the various genomic variants of SARS‐CoV‐2 prevalent today and the efficacy of the various vaccines in use against them. Re‐infection risk is a particular concern in patients with immunodeficiency due to their inability to mount sufficient protective antibody response and may have a heightened risk for COVID‐19 re‐infection. We report one such case of rituximab‐induced acquired immunodeficiency, with documented hypogammaglobulinaemia, who had a recurrent COVID‐19 infection.

## CASE REPORT

**FIGURE 1:**
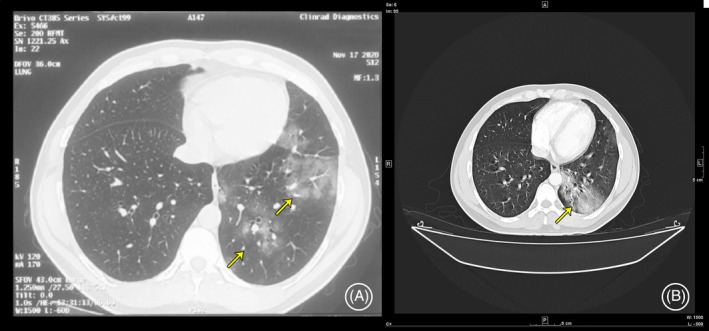
Computed tomography thorax in (A) November 2020 and (B) February 2021 (A, left lower lobe evolving consolidation; B, persistent left lower lobe consolidation)

A 35‐year‐old gentleman came to our institution in February 2021 for evaluation of persistent fever and non‐resolving pneumonia. He had a polymerase chain reaction (PCR)‐confirmed SARS‐CoV‐2 infection in October 2020, with classical radiological findings. He was managed conservatively under home quarantine. Two weeks later (November 2020), he had a recurrence of high spiking fever. Imaging revealed a left‐sided lung consolidation. He was treated for probable pneumonia with oral amoxicillin‐clavulanate. At 1‐month follow‐up (December 2020), there was an inadequate clinical improvement with an increase in the size of the consolidation (Figure 1). Bronchoscopy was done, and bronchoalveolar lavage grew *Streptococcus pneumoniae*. He received another course of linezolid antibiotic.

In 2017, he had probable vaccine‐related/autoimmune optic neuritis for which he received 13 doses of rituximab between January 2017 and May 2019 (600 mg per dose). He had developed rituximab‐induced hypogammaglobulinaemia in 2019. Since then, he has had persistent B‐cell depletion and low immunoglobulin levels (Table 1). The absolute B‐cell count was zero.

**TABLE 1:** Persistent IgM deficiency

Immunoglobulin	November 2019	February 2021	June 2021	Reference range (mg/dl)
IgG	930	839	898	700–1600
IgA	117	117	71	70–400
IgM	27	12	20	40–230

During our evaluation, SARS‐CoV‐2 real‐time reverse transcription PCR (RT‐PCR) was negative (February 2021 and March 2021). Antibodies to SARS‐CoV‐2 nucleoprotein (N) and spike receptor‐binding domain (S‐RBD) tested on the Roche Elecsys platform were undetectable in February 2021. We considered the following differentials: tuberculosis (TB), organizing pneumonia, lymphoma, Antineutrophil Cytoplasmic Antibodies (ANCA) associated vasculitis and lung malignancy. Sputum Xpert‐TB‐PCR was negative. Multiple blood cultures, bone marrow biopsy and culture reports for routine bacteria, Mycobacterium and fungal organisms were negative. A bronchoscopic transbronchial lung biopsy was done. Histopathology was suggestive of organizing pneumonia. Past COVID‐19 infection was considered as the likely cause for organizing pneumonia. He was started on high‐dose steroids, with which he improved.

**FIGURE 2:**
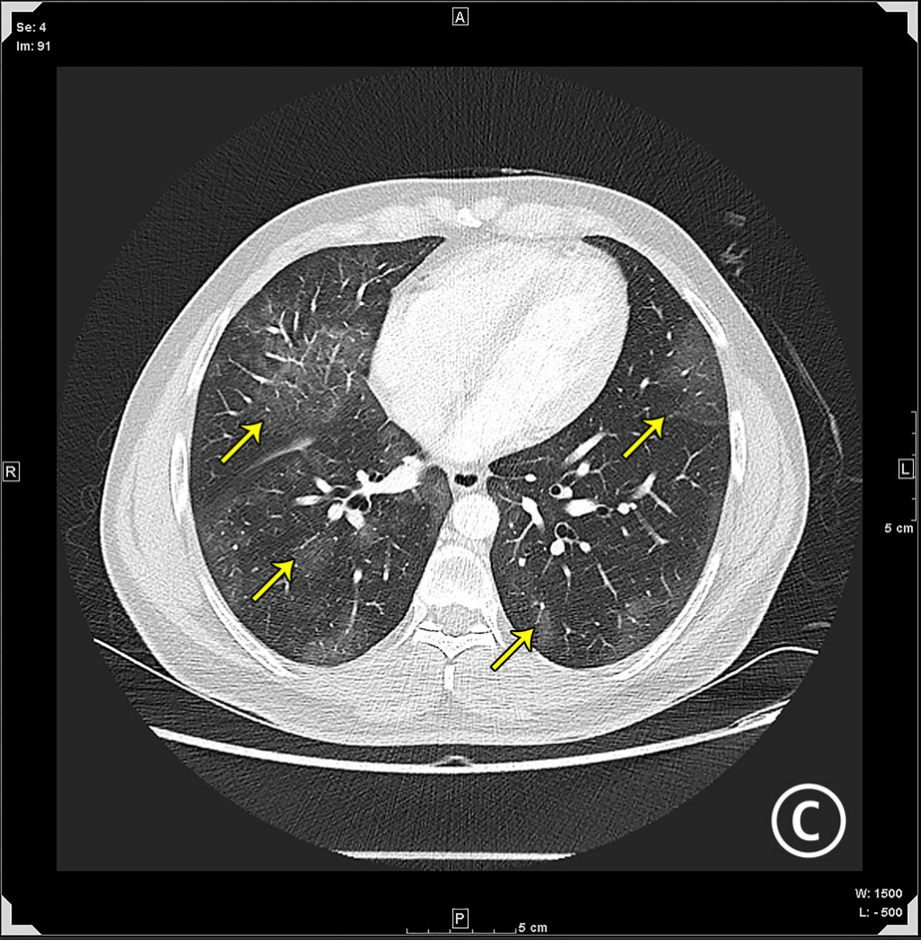
Computed tomography thorax in April 2021 shows bilateral new‐onset ground‐glass opacities

He was readmitted in April 2021 with a persistent fever. Since the patient was on high‐dose steroids, the fever was attributed to a probable secondary infection or organizing pneumonia itself. Sputum culture grew *Pseudomonas aeruginosa*, and an antibiotic, piperacillin, was commenced. Computed tomography thorax showed resolution of the previously seen left lower lobe consolidation but had new‐onset bilateral ground‐glass opacities (GGOs) (Figure 2). Nasal swab for SARS‐CoV‐2 RT‐PCR was positive (Cepheid assay, April 2021), with a Ct value of Envelope gene (E gene) 23.2 and nucleoprotein gene (N) 24.1. A diagnosis of COVID‐19 re‐infection was made. He received remdesivir injection and other symptomatic medications. He became afebrile within 2 days and was discharged from the hospital. During routine follow‐up in June 2021, he was asymptomatic and was now positive for antibodies to SARS‐CoV‐2 (anti‐N: 9.6 and anti‐S‐RBD: 41.4) (Table 2).

**TABLE 2:** Serum antibody levels before and after the second COVID‐19 infection in April 2020

Roche serum antibody test	February 2021	June 2021	Reference range (U/ml)
Roche Anti‐S	0.4	41.36	<0.8
Roche Anti‐N	0.036	9.61	<0.8

## DISCUSSION

Rituximab is a monoclonal antibody against CD20, a cell surface antigen for B cells.^2^ With rituximab‐induced B‐cell depletion, the IgG and IgM production and blood levels drop in a dose‐related fashion. IgG antibody levels recover within a year in most cases after stopping rituximab therapy. IgM deficiency, on the other hand, recovers slower than IgG. Rituximab‐induced B‐cell depletion usually leads to insufficient humoral response to viral infections. Patients on rituximab therapy tend to have lower or absent SARS‐CoV‐2 antibody levels than those who discontinued rituximab.^3^, ^4^ It is likely that any immunodeficiency—primary or acquired—will result in poor antibody response to natural infection or vaccine, leading to re‐infections.

In our case, the first infection was a documented PCR positivity in October 2020, and SARS‐CoV‐2 antibody status (anti‐N and anti‐S‐RBD) was found negative in February 2021, which may be explained by either a non‐response or waning of antibody levels. Re‐infection occurred in April 2021 with the onset of a new lung lesion that was managed appropriately. He mounted a robust antibody response to the second infection.

Váncsa et al. analysed 123 cases of recurrent COVID‐19 and reported a mean duration of 47.9 ± 30.1 days between the primary and the recurrent SARS‐CoV‐2 positivity. The proportion of patients with the second positive test 60 days later were few.^5^ A review by Long et al. analysed all published reports of re‐infection and suggested that most cases found positive on repeat PCR within 28 days of the primary infection are likely due to detection of residual viral RNA from the initial infection due to the use of highly sensitive real‐time PCR assay with a lower limit of detection of ~10 copies of viral RNA/ml. However, if the time gap was more than 3 months, the chances of the repeat positivity being due to re‐infection were high.^6^ Our patient had a 5‐month gap between positive PCR tests. Furthermore, two negative RT‐PCR tests in March and April 2021 effectively rule out the persistence of viral RNA from the original infection. The lack of SARS‐CoV‐2‐specific immunity in February 2021, either due to waning or absence of antibody production and a subsequent positive RT‐PCR (April 2021) with demonstrable antibody positivity in the post‐infection serum sample, confirms the second episode to be a re‐infection.

The prevalence of true re‐infections is likely to be around 1%.^7^ The case fatality rate among those who were re‐infected was not alarmingly high. While most cases of re‐infection have a mild clinical course,^5^, ^6^ in our case, the repeat episode also presented as a severe disease with a new‐onset GGOs—classical lung features of COVID‐19. There is growing evidence of repeat infections occurring in immunocompromised who do not mount an antibody response, being more severe than the primary infection.^8^, ^9^ This is an important consideration for special populations with significant co‐morbidity and/or immunodeficiency, where natural infection or vaccination may not provide sufficient immunity to re‐infection. The CDC now recommends a third COVID vaccine booster dose for patients with immunodeficiencies.^10^, ^11^, ^12^

## CONFLICT OF INTEREST

None declared.

## AUTHOR CONTRIBUTION

Jefferson Daniel wrote the manuscript. Balamugesh Thangakunam, Barney Thomas Jesudason Isaac, Mahesh Moorthy and Devasahayam Jesudas Christopher were involved in interpreting the data and provided expertise and feedback. All authors revised the manuscript critically and approved the final version before submission.

## ETHICS STATEMENT

The authors declare that appropriate written informed consent was obtained for the publication of this case report and accompanying images.

## Data Availability

Data available on request due to privacy/ethical restrictions.
